# Parathyroid carcinoma: epidemiology and genetics

**DOI:** 10.1530/EO-25-0104

**Published:** 2026-08-14

**Authors:** Daniela Betea, Patrick Petrossians

**Affiliations:** Department of Endocrinology, Centre Hospitalier Universitaire de Liège, University of Liège, Liège, Belgium

**Keywords:** parathyroid, primary hyperparathyroidism, parathyroid carcinoma, hypercalcaemia, malignancy, epidemiology, genetics

## Abstract

Primary hyperparathyroidism (PHPT), considered a rare disease almost a century ago, has shown a notable increase in incidence over the past five decades, driven largely by improved diagnostic screening and greater clinical awareness. Following the same trend, the incidence of parathyroid carcinoma (PC), a very rare but aggressive malignancy accounting for approximately 1% of PHPT cases, appears to be rising. This increase parallels the overall rise in PHPT detection, suggesting that enhanced surveillance contributes to identifying more PC cases. PC is mostly characterized by severe hypercalcaemia, due to tumoral parathyroid hormone (PTH) over-secretion and a high rate of local recurrence and metastasis, making early diagnosis and surgical treatment crucial. Recent advances in genetics and epigenetics have profoundly expanded our understanding of PC pathogenesis. Germline and somatic mutations in the tumour suppressor gene *CDC73* (encoding parafibromin) are implicated in up to 75% of familial and sporadic PC cases. Additional recurrent mutations in genes such as *MEN1* and *PRUNE2*, alterations in cell cycle regulators such as *CCND1* and *EZH2*, and disruptions in signalling pathways including PI3K/AKT/mTOR have been identified. Epigenetic modifications, including microRNA expression changes and promoter hypermethylation, also play a significant role in tumour development and progression. This mini review synthesizes epidemiological trends demonstrating an increasing incidence of PC following the rise of PHPT and highlights pivotal genetic and epigenetic findings contributing to tumourigenesis. Integrating new molecular insights with advances in clinical surveillance offers promise for improved diagnosis, prognostic stratification, and the development of future targeted therapies for parathyroid carcinoma.

## Introduction

Primary hyperparathyroidism (PHPT) is one of the three most common endocrine disorders and is frequently encountered in both general and specialist practice settings. Incidence estimates for PHPT have varied quite widely over the past five decades, which has been due to study methodologies. The most recent population-based data on PHPT report an incidence range of 48.3–50.4 cases per 100,000 persons/year in the US (1). The incidence increases in an age- and sex-related manner, the ratio being close to 1 for people aged < 40 years and twice as common in females as in males over the age of 75. The peak incidence is in women aged between 50 and 60 years (2). Due to increased diagnostic accuracy and rising life expectancy, the overall age-adjusted prevalence rate of PHPT in the US in 2010 was estimated at 233/100,000 in women and 85/100,000 in men, with 70,000–90,000 new cases diagnosed each year (2, 3, 4). The incidence in the general population in Western Europe showed the same trend as in the US. The incidence of PHPT increased to 40.3 per 100,000 women/year and 13.7 per 100,000 men/year (5). The incidence increased in an age-related manner from 0.59 per 10,000 persons/year in those aged < 30 years to 12.4 per 10,000 persons/year in those aged >70 years. PHPT is 2.5 times more frequent in women than in men (6).

PHPT is considered the third most frequent endocrinopathy after thyroid disorders and diabetes mellitus. PHPT usually presents as a single parathyroid adenoma (PA) in approximately 80% of cases, multiglandular disease in 15–20% of cases, and parathyroid carcinoma (PC) in <1% of cases. PC is the rarest endocrine malignancy, representing less than 0.005% of all malignancies worldwide (7). The first description of a metastatic, non-secreting parathyroid carcinoma was reported by De Quervain in 1904 (8), followed by the description of a secreting parathyroid carcinoma by Sainton in 1933 (9). Since then, PC has generally been described in single case reports and small series from tertiary referral centres (<50 patients). Underlining the extreme rarity of PC, only about 1,000 patients had been described up to 2004 (10). In the past two decades, however, larger series of PCs from national databases, cancer registries, and multicentre collaborative studies have increased that number to around 3,000 described cases worldwide (11, 12, 13, 14, 15).

This mini review aims to briefly review the evolution in epidemiology of parathyroid carcinoma and in the recent advances in aetiology and pathogenesis of the disease.

## Incidence and prevalence in parathyroid carcinoma

The earliest reports from individual tertiary referral centres estimated that PC accounted for less than 1% of all cases of PHPT in series from the US and Europe (11, 16, 17, 18, 19, 20, 21, 22, 23). Two single-centre series from Italy and Japan reported a higher rate of 5% of PC in PHPT, although the higher rate may have been due to more inclusive diagnostic criteria (24, 25).

### National and international databases

**Figure 1:**
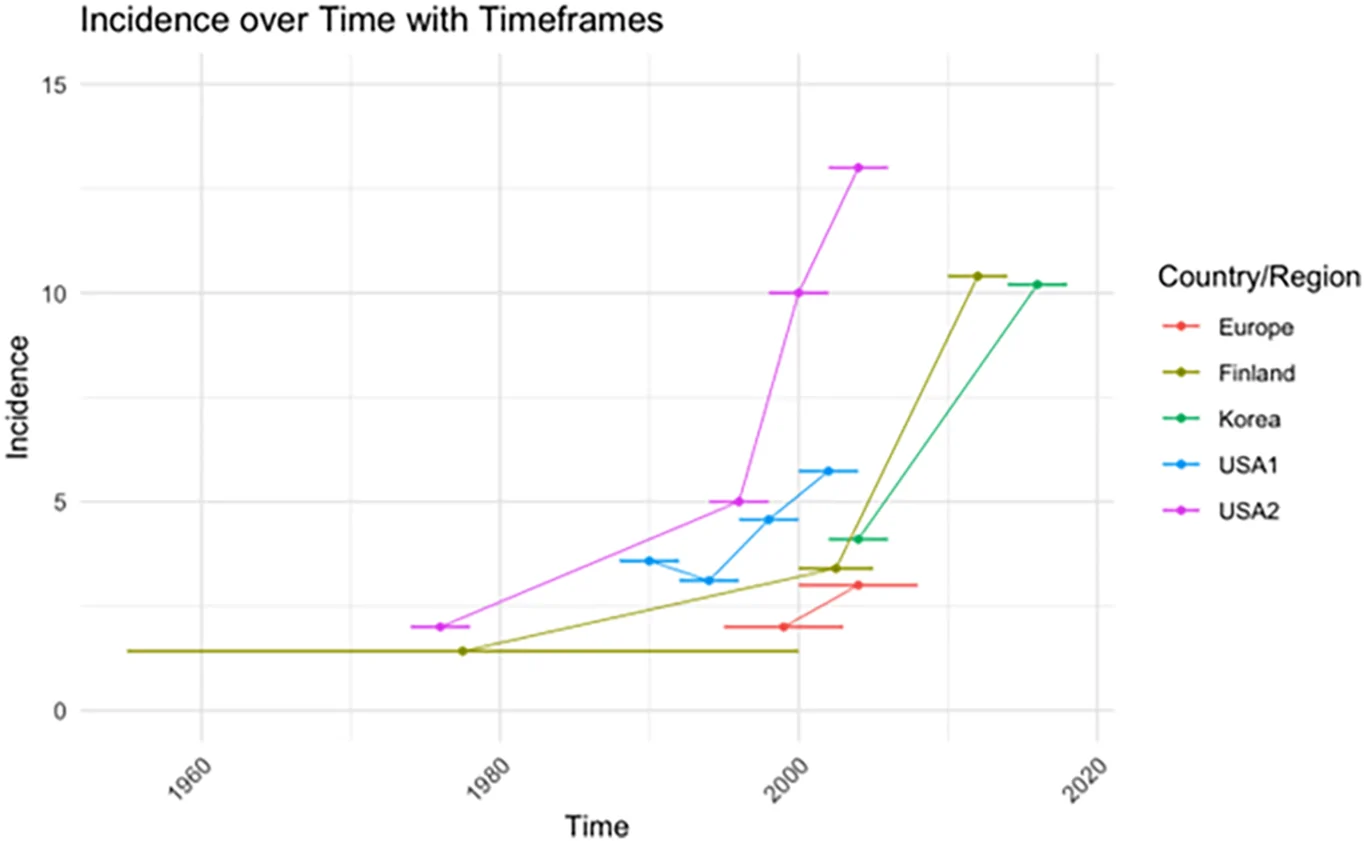
Incidence rates of parathyroid carcinoma among selected regions over time: USA1 (13), USA2 (26), Europe (28, 29), Finland (30) and Korea (37). The horizontal bars represent the different time periods of observation used in each study since 1955.

Much of our understanding of the epidemiology of PC comes from studies using the data from Surveillance, Epidemiology and End Results (SEER) national cancer registry in the US. Studying an early period (1988–2003), Lee *et al.* reported epidemiological data in 224 patients with PC (13). Over the 16-year period studied, the incidence of PC increased by 60% from 3.58 to 5.73 per 10,000,000 persons. The authors hypothesized that changes in the 2002 NIH Consensus guidelines for parathyroidectomy in asymptomatic PHPT may have resulted in increased identification of asymptomatic PC through increased serum calcium screening and consequent referral to surgery/pathology (Fig. 1).

A second SEER study, assessing PC trends over a longer period (1973–2014), identified 520 patients (26). The initial incidence was 2/10,000,000 persons (1974–1977), then 5/10,000,000 persons (1994–1997), increasing to 10/10,000,000 persons (1998–2001); the rate stabilized at 13 cases/10,000,000 persons from 2002. The increasing incidence was attributed to detection of patients with locoregional disease and tumour size < 3 cm. The median age at diagnosis was 56 years, and the sex distribution was equal. More recent diagnosis, young age, female gender and the absence of distant metastasis were the main factors significantly associated with an improved overall survival rate. That study confirmed that the previously observed increase in incidence might be due to improved screening and referral to surgery for PHPT. The time of the guideline changes mirror the changing incidence in PC, rising from 1994 to 2005, reaching a plateau and remaining stable thereafter (Fig. 1).

The increased incidence trend was confirmed further by the largest cohort, extracting data from the same SEER database (1975–2016), selecting 609 cases, and investigating demographic, clinical and pathological factors affecting prognosis and survival of patients with PC. The overall cohort from 1975 to 2016 revealed an annual percentage change of +6% (27). The mean age at diagnosis was 62, and only 14% of patients were diagnosed at younger than 40 years. There was a slightly higher incidence in men (52%) and most patients were Caucasian (75%).

The RARECARE project was a population-based study from 89 cancer registries in European Union (EU) countries that studied cancer patients diagnosed from 1978 to 2002. This database reported 176 cases of PC between 1995 and 2002. The estimated incidence was 2–3/10,000,000 persons/year which led to an estimated 109 cases annually in the EU. The regional rates were similar across countries of the EU (28). More recent data extracted from RARECARE involved 117 cancer registries from EU participating countries, were published five years later and covered a longer period between 1978 and 2007. The reported incidence was 3/10,000,000 persons/year, with 410 observed cases between 2000 and 2007 (29) (Fig. 1).

The same significant increase in the incidence of PC in Europe overall was reported in a nationwide study from Finland. In the early period (1955–2000), the mean incidence was 1.42 cases/10,000,000 persons (range 0.52–2.14). During the latest period (2000–2013), the incidence rose from 3.4 (2000–2004) to 10.4/10,000,000 persons/year in 2010–2013 (30) (Fig. 1).

Other studies from Europe were published over the past two decades: a nationwide study from the Netherlands, extracting data from the National Cancer Registry (31), followed by multicentre studies from Spain with 35 centres (32), Germany, Austria and Switzerland with 29 centres (the NEKAR study) (33), and Italy with 24 centres (pTIRANI Study) (34). These studies reported a lesser increase in incidence (from 1 to 1.5%) and similar demographic findings regarding age and gender. PC remains a very rare cancer, without a sex-related predominance, usually diagnosed in the fifth decade of life as reported previously (14). Patients with PC are relatively younger (mean age ca. 50 years) than patients with benign PHPT (35).

Sustaining the previous findings, a recent single centre Australian study from Sydney University reported trends over a 52-year period (1958–2010). Over the study, a total of 3,851 patients underwent surgery for parathyroid disease: there were 3,306 cases of benign adenoma, 524 cases of hyperplasia and 21 cases of PC. The overall incidence of PC for this series was 0.5%. Among the 21 cases with confirmed PC, only three cases were reported in the first 30 years of the study with almost half of cases (*n* = 11) diagnosed in the past 5 years (36). The mean age at the first presentation was 52 years (range 19–74 years), and 12 patients (57%) were male.

In Asia, a nationwide retrospective study from Korea using data from the National Health Insurance Service (NIHS) from 2002 to 2017 was recently published. A total of 255 patients with PC were analysed. The same trend in increasing incidence over time was observed. The incidence was 3.8/10,000,000 persons/year in 2003, rising to 6.6/10,000,000 persons/year in 2016. The mean age at diagnosis was 53 years and there was a sex imbalance with more females (*n* = 155 (61%) than males (*n* = 100; 39%) (37) (Fig. 1).

Two studies from China were recently reported. In the first study, a large cohort of 234 PC cases from 60 teaching hospitals was extracted from three major databases across China, covering a period between 1984 and 2015. The same increase in the number of PC was found elsewhere, from 13 cases identified between 1996 and 2000, to more than 100 cases identified between 2011 and 2015. The male:female ratio was almost 1:1 (120 males and 114 females). The median age at diagnosis was 45.5 years. The study outlined that a large proportion (191 cases) of patients were misdiagnosed prior to surgery (38). The second study was from a single tertiary centre with 126 patients who underwent surgery from 2010 to 2023. The reported incidence was related specifically to distant metastasis (DM). The cumulative incidence was 14, 34 and 67% at 5, 10 and 20 years following diagnosis (39).

Two single-centre studies from Japan did not find the higher incidence reported in the early study from Obara and Fujimoto (18). The first study reported 38 patients treated from 1981 to 2005. The incidence of PC among cases of PHPT was 2.8% in this series. There were 17 men and 21 women with a median age of 51 years at initial parathyroid surgery (40). The second study included 38 patients who received surgical treatment from 1979 to 2020, diagnosed among 1,521 patients with PHPT, with an incidence of 2.5%. There were 11 men and 27 women and the mean age at surgery was 56.5 years (41).

Few reports come from the Indian subcontinent and no specific data regarding incidence were available. One series reported 10 cases of PC among 245 PHPT (4.08%) treated in a single tertiary centre from 1988 to 2008 (42). Another study reported 19 cases of PC among 112 PHPT treated from 2010 to 2018 in a single tertiary centre. The mean age at presentation was 46 years. There was no gender disparity, with 53.3% of patients being male (43).

Data regarding other continents such as South America and Africa were scarce (44).

The global population has grown significantly since the mid-20th century, from approximately 2.5 billion in 1950 to over 8 billion. While this growth increases the absolute number of individuals at risk, the incidence of PC remains low. The observed increases in certain regions seem to be more closely linked to improved healthcare infrastructure, routine biochemical screening, and heightened clinical awareness rather than population growth alone or increases in life expectancy.

## Diagnostic challenges and the issue of overdiagnosis

The rise in incidence of PC over time in the studies outlined should be viewed with certain caveats. PC can be diagnosed in a number of clinical settings, some of which are unequivocal (presence of metastases) and other less so. Diagnosing PC can be practically challenging, and relying solely on limited pathological features, such as the involved resection margins, mild fibrosis and limited adhesion to local tissues, could lead to misclassification of benign or atypical parathyroid tumour as PC. The issue of avoiding overdiagnosis of PC has been addressed in a number of studies, as recently outlined by Juhlin and Mete (45). They highlight the importance of combining the clinical features of the parathyroid lesion (e.g. size, severity of hypercalcaemia, peak PTH levels) with knowledge/avoidance of known morphological pitfalls (biopsy and mechanical artefacts, peliosis, ectopic parathyroid tissue and nodular/hyperplastic changes in secondary hyperparathyroidism), in addition to a comprehensive use of genetic and molecular biomarker data, including but not limited to parafibromin staining. To strengthen the rigour of the diagnostic approach around PC to avoid potential overdiagnosis, certain key practical steps have been suggested. These relate to the multidisciplinary management of patients with PHPT including endocrinology, genetics, surgery and, to a large extent, pathology.

- **Clinical factors**: Severe hypercalcaemia, markedly elevated PTH and large parathyroid mass raise high suspicion. Metastasis is the only definitive characteristic in the diagnosis of PC.
- **Pathological factors:**
  - **Histology**: Invasion into local organs or vascular, lymphatic or perineural invasion is diagnostic of PC.
  - Presence of fibrous bands, coagulative necrosis, and a high mitotic index support a PC diagnosis. Their presence alone is insufficient to diagnose PC.
  - **Immunohistochemistry**: Absent parafibromin staining, potentially with loss of RB, or APC, and a high Ki-67 index strongly supports a PC diagnosis.
  - Comprehensive analysis of the entire resected tumour at various levels may be needed.
- **Genetic factors:** Germline/somatic *CDC73* sequencing for pathogenic/likely pathogenic variants provides valuable supportive information.

## Aetiology and molecular pathogenesis

The aetiology of PC remains incompletely understood, but recent years have seen both sporadic and hereditary factors becoming implicated. Most cases occur sporadically, and only a subset is associated with genetic/familial syndromic forms. Environmental or lifestyle factors have not been clearly established, and unlike other endocrine tumours, there is limited evidence linking radiation exposure or long-standing end-stage kidney disease to its development (46). The rarity of the disease has contributed to gaps in identifying definitive causative mechanisms.

In the past two decades, the identification of distinctive genetic alterations, epigenetic changes and molecular signatures, characterizing parathyroid malignancies, represents a major advance in the identification of potential diagnostic biomarkers that can clearly distinguish PCs from benign parathyroid tumours and provides potential therapeutic targets for these malignant neoplasms.

PCs occur generally as a sporadic, single-gland tumour; in rare cases, PCs manifest as a part of specific hereditary syndromic or non-syndromic diseases. The majority of PCs are functioning tumours with over-secreting PTH, which is responsible for the clinical picture of PHPT and hypercalcaemia. In only 2% of cases, non-functioning PC variants occur, with normal serum calcium and PTH values, manifesting with symptoms of local invasion and compressive signs (47, 48).

PCs have a very heterogeneous behaviour, exhibiting slow, indolent progression in some cases and a rapid course in others, associated with severely elevated PTH and severe, frequently intractable hypercalcaemia. The latter represents the major cause of death in most cases of PC. The overall survival rate is 78–85% and 49–70% at 5 and 10 years after diagnosis, respectively (12, 13, 22, 49). At the first presentation, 10–30% of patients with PC already have distant metastases, most commonly in the lungs, bone or liver (12, 50, 51).

The only curative treatment is surgery. The absence of specific clinical, biological or imaging techniques that can distinguish malignant from benign lesions at first presentation makes a clear pre-surgical diagnosis extremely difficult. Since PCs require an oncological surgical approach, with *en bloc* gland resection and clear operative tissue margins, a misdiagnosis may lead to inadequate surgical resection and consequently to a high recurrence rate and risk of distant metastases. More than 50% of patients with PC present with metastases following an initial diagnosis of benign disease, with a consequent severe impairment of their prognosis and reduction of overall survival (52, 53). The diagnosis of malignancy can occur intraoperatively or postoperatively with histological confirmation, or frequently, due to local relapse or distant metastases during prolonged follow-up.

## Genetic studies

PCs can occur as sporadic single-gland disease in the absence of known hereditary factors, or infrequently as part of a known syndrome. Hyperparathyroidism-jaw tumour (HPT-JT) syndrome is a rare condition associated with PHPT, ossifying fibroma of the mandible and maxilla, and tumours of the kidney and uterus (54). Parathyroid tumours are the first manifestation in more than 90%, with 15% being PC. The usual presentation is a solitary parathyroid tumour, but multiglandular involvement may be present in 15% of cases (54, 55, 56). PCs can also occur rarely in familial isolated primary hyperparathyroidism (FIHP) (57, 58). Extremely rare cases of malignant PCs have been reported in multiple endocrine neoplasia type 1 (MEN1) syndrome (<15 cases) (59, 60) and in multiple endocrine neoplasia type 2A (MEN2A) syndrome (only three cases) (61) (Table 1).

**Table 1:** Molecular mechanisms implicated in parathyroid carcinoma.

Gene and chromosome location	Mutations	Encoded proteins	Frequency in PC
*CDC73* Tumour suppressor 1q31.2 (59, 72)	Biallelic inactivating mutations Gene loss	Parafibromin	70–90% mutations in sporadic PC 50–55% 1q31.2 LOH in sporadic PC 50–75% mutations in HPT-JT families
*MEN1* Tumour suppressor (88, 91, 92)	Germline/somatic (frameshift, nonsense, missense)	Menin	<15 MEN1 cases 45% germline, loss-of-function mutations 13–37% somatic alteration in MEN1 gene in sporadic PC
*RET* Oncogene 10q11.21 (62, 72)	Germline activating mutations	Receptor protein kinase	3 cases with metastatic PC
CCND1 Protooncogene 11q13.3 (95)	Somatic cyclin D1 gene copy amplifications	Cyclin D	29% of patients: gene amplification 60–90% of cases cyclin D1 overexpression in sporadic PC
*PRUNE2* Tumour suppressor 9q21.2 (95, 108)	Germline and/or somatic biallelic inactivating mutations Gene loss	Prune homolog 2	20% of sporadic PC
*EZH2* Protooncogene 7q.36.1 (102)	Somatic gene copy amplification	EZH2 histone	60% of sporadic PC
*APC* Tumour suppressor 5q22.2 (79, 106)	Loss of APC expression Hypermethylation of APC promoter	Adenomatous polyposis coli protein	75% of sporadic PC >85% in sporadic PC

Hereditary and syndromic forms of PC can be associated with germline inactivating variants in the tumour suppressor genes: *CDC7*3 gene (in HPT-JT syndrome) and *MEN1* gene (in MEN1 syndrome). For the *RET* oncogene variants (MEN2A syndrome), which are activating, only one copy of the mutated gene is required for tumour development. No genotype–phenotype correlation has been found to explain why these few individuals develop PCs instead of PAs, which typically affect patients with MEN1 (62).

### *CDC73* (cell division cycle 73) gene

The *CDC73* gene, comprising 17 exons, is located on the chromosome 1q31.2 and encodes a nuclear protein of 531 amino acids, *parafibromin*, a polymerase-associated factor 1 (PAF 1)/RNA polymerase II complex component, involved in genetic transcription control pathways and chromosome remodelling (63). It was first identified in association with HPT-JT kindreds by Carpten *et al.* (64). Inactivating variants (80% of *CDC73* pathogenic variants in PC) predict a truncated parafibromin protein or loss of protein transcription and loss of tumour suppressor activity.

In sporadic PC, somatic *CDC73* pathogenic variants were first reported in 2003 by Howell (65) and Shattuck (66) and subsequently confirmed by others (67). Biallelic somatic inactivation of the *CDC73* gene has been reported in 40–60% of sporadic PCs in some studies, while others have found even higher rates depending on the population and detection methods (65, 66, 67, 68, 69, 70, 71). Most of these variants are nonsense and predict a loss of or reduced parafibromin expression. In about 20–40% of patients with apparently sporadic PC, the *CDC73* variants are germline, suggesting variable penetrance, or patients might have underdiagnosed HPT-JT syndrome, where the family history is unknown, or a *de novo* variant case of mosaicism (66, 72, 73, 74). Loss of heterozygosity (LOH) at the *CDC73* locus is reported in 50–55% of sporadic PC cases (72).

In hereditary PC, heterozygotic inactivating variants of *CDC73* gene have been detected in 50–75% of HPT-JT families and in about 8% of FIPH families (73, 75). Moreover, the same germline variant may be associated with HPT-JT, FIHP and sporadic PC in different patients (56, 64). There is no genotype–phenotype correlation, but the underlying mechanism for clinical disease variability is unknown (63, 65, 66, 72). In 25% of HPT-JT families with no variant detected, a *CDC73* gene deletion or epigenetic silencing of *CDC73* by hypermethylation of the promoter region (described as a somatic event) could occur (76, 77). The pattern of transmission is autosomal dominant, and genetic counselling is always important.

Immunohistochemistry (IHC) showing the complete absence of nuclear parafibromin immunoreactivity in all tumour cells identifies a ‘parafibromin-deficient’ tumour, which is highly correlated with an underlying *CDC73* gene variant, supporting the diagnosis of malignancy. It has a reported sensitivity of 75–90% and specificity of 90%. Loss of parafibromin staining is a useful potential prognostic factor that is an indicator of higher risk of recurrence, metastasis and mortality in PC (78). Atypical staining patterns with focal nuclear loss or selective nucleolar loss have been described in tumours with specific *CDC73* variants (79, 80). However, it is important to highlight that parafibromin may be a difficult stain to perform and interpret and this tool should never be used alone to discriminate PC from atypical parathyroid tumour or PA (79, 80, 81).

### *MEN1* (multiple endocrine neoplasia type 1) gene

MEN1 is a rare, highly penetrant, autosomal dominant endocrine disorder caused by inactivating germline pathogenic variants in the tumour suppressor gene *MEN1*, located on chromosome 11q13, which encodes *menin*, a 610 amino acid protein. Menin has roles in transcription regulation, cell division and proliferation (82, 83). More than 90% of tumours in patients with *MEN1* will have LOH of the *MEN1* gene, with loss of menin expression and loss of its tumour suppressor role (84).

MEN1 classically combines PHPT, pituitary and pancreatic islets tumours, although other rare MEN1-associated tumours have been described (85). Parathyroid tumours are often the first disease manifestation and PHPT has the highest lifetime penetrance of the component MEN1 tumours, occurring in 95% of MEN1 patients (52 and 100% penetrance by the ages of 20 and 60 years, respectively) (86). Parathyroid tumours in *MEN1* are benign in 99% of cases, but many MEN1 patients with PHPT have multiglandular disease. Even though PHPT is the most penetrant disease manifestation in MEN1, only a tiny minority go on to develop PC. In total, less than 15 cases have been reported in the setting of MEN1 to date. About 60% of these patients have local invasion or distant metastasis (73, 87, 88).

Outside of the settings of MEN1, about 40–50% of PC have LOH of chromosome 11q (the location of the *MEN1* gene) and more than 35% of PC have combined LOH of 11q and 1q (location of *CDC73* gene), suggesting a general contribution of *MEN1* gene loss in PC pathogenesis (89, 90, 91, 92).

### *RET* gene (rearranged during transfection) gene

*RET* is an oncogene activated by DNA rearrangement, located on chromosome 10q11.21. The *RET* gene encodes a receptor tyrosine-protein kinase involved in cell proliferation, migration and differentiation, neuronal navigation, following binding of glial cell-derived neurotropic factor (GDNF) ligands. In the absence of ligand, RET can trigger apoptosis.

MEN2 (Sipple syndrome) comprises three variants: MEN2A, MEN2B and familial medullary thyroid carcinoma (FMTC). MEN2A is associated with medullary thyroid carcinoma (MTC), pheochromocytomas and parathyroid tumours, which are present in > 99%, almost 40 and 30% of patients, respectively (93). MEN2B is associated with MTC, pheochromocytoma and mucosal neuromas, intestinal autonomic ganglion dysfunction and Marfanoid status (85). In the third variant, MTC is the only manifestation. In this syndrome there is a genotype–phenotype correlation between *RET* variants and the three clinical manifestations (94).

Only three PC cases have been reported in patients with MEN2A. All patients were male, and all had metastatic PC at diagnosis. In two variants, the germline or somatic origin was not defined and the third was a germline variant (62).

### *PRUNE2* gene (prune homolog 2) gene

*PRUNE2* is located on chromosome 9q21.2, encodes a 3088-amino acid protein that regulates cell differentiation and survival by suppression of Ras homolog family member A (RhoA) activity, which results in inhibition of oncogenic cellular transformation. The inactivation/loss of the protein could play a role in promoting malignant carcinogenesis and tumour progression. Germline and somatic variants in *PRUNE2* were identified in 2015 (70). They were associated with PC in 18% of cases studied: one germline variant and two somatic variants in PCs without *CDC73* variants and two nonsense somatic variants in an individual PC with a *CDC73* variant. These findings suggest that *PRUNE2* variants could contribute to the development of sporadic PCs (70, 95).

### *CCND1* (cyclin D1) gene

*CCND1* (previously termed the *parathyroid adenoma 1* (*PRAD1*) gene) is located on chromosome 11q13.3 and encodes a 295 amino acid protein, *cyclin D1*, which is a component of the cyclin D1-cyclin-dependent kinase 4 (CDK4) complex. It is a cell cycle regulator promoting G1/S phase transition. The overexpression of cyclin D1 has been reported in 65–90% of PC (96, 97), being associated with PC cell proliferation and a Ki-67 index of >5% (90). *CCND1* overexpression is due to the somatic amplification of the gene in 70% of sporadic PC, which is more prevalent than in PA (20%) (98). Amplification of *CCND1* and somatic loss/inactivation of *CDC73* are usually mutually exclusive events in the same tumour (95, 99).

### *EZH2* (enhancer of zeste 2 polycomb repressive complex 2 subunit) gene

The *EZH2* gene is located on chromosome 7q36.1 and encodes a 746-amino acid histone methyltransferase enzyme that directly controls gene methylation and transcriptional repression (100). *EZH2* gene variants are very rarely found in PC (101), but amplification of the *EZH2* gene has been reported in 60% of PC, 20% of PA and 50% of parathyroid hyperplasia (102). *EZH2* may interact with β-catenin, inducing activation of Wnt/β-catenin signalling, and increasing the expression of its target gene *CCND1* (103).

### *APC* (adenomatous polyposis coli) gene

*APC* is a tumour suppressor gene located on chromosome 5q22.2, encoding a 2843-amino acid protein, which controls β-catenin ubiquitination and proteolysis, inhibiting canonical Wnt signalling. Although *APC* variants have not been observed, loss of APC expression was reported in 75% of PCs, whereas it was maintained in 100% of PA (70, 79, 104, 105). Alteration of APC expression in PC may involve hypermethylation of the *APC* promoter, as its methylation levels were found to be significantly higher in PC (>85%) than in normal parathyroid glands (>15%). Treatment of cultured PC cells with the DNA methylation inhibitor 5-aza-2′-deoxycytidine (decitabine) resulted in re-expression of *APC* mRNA, APC protein and reduced cell viability. It was suggested that decitabine could be an additional option in the treatment of patients with recurrent or metastatic PC (106).

### PI3K/AKT/mTOR signalling pathway

The PI3K/AKT/mTOR pathway is a critical intracellular signal transduction pathway involved in the regulation of cell metabolism, growth, proliferation, apoptosis and angiogenesis. Dysregulation of this signalling pathway is involved in the onset and development of multiple types of malignancies, including endocrine neoplasms, being associated with poor prognosis and treatment resistance (107).

Genomic profiling studies have revealed the presence of somatic variants in gene encoding components of this signalling pathway in 30–80% of primary and recurrent/metastatic PCs, including those affecting *PIK3CA*, *TP53*, *MTOR*, *PTEN*, *TSC1* and *TSC2* (92, 95, 108, 109, 110, 111). Results from these studies suggest that altered PI3K/AKT/mTOR pathway activity, rather than a single gene, could be a major oncogenic factor in parathyroid carcinogenesis. A recent report performed comparative analysis of clinical characteristics and outcomes in 64 patients with different variant types of PCs (66 tumours). PI3K/AKT/mTOR activating variants indicate a more severe clinical profile with significantly poorer overall survival outcomes, which was not observed in those with *CDC73* variants (112).

Another whole-genome sequencing study found somatic variants in the PI3K/AKT/mTOR pathway in 64% of cases (9/14), showing a positive correlation with cancer recurrence/metastasis (111).

Thus, the use of mTOR inhibitors could represent an effective therapy in patients with PC having these variants. One study reported therapy with everolimus an mTOR inhibitor in association with vandetanib, an anti-angiogenic tyrosine kinase inhibitor in a metastatic PC case with two somatic variants in TSC1 gene, a known regulator of PI3K/AKT/mTOR pathway (109).

In a 19-patient series of recurrent/metastatic parathyroid carcinoma, 47% harboured at least one potentially actionable alteration in *ROS1, PTEN, TSC1* and *PIK3CA*, indicating that nearly half of advanced cases may qualify for rational targeted approaches with tyrosine kinase inhibitors (108).

## Other genetic pathways and actionable driver mutation

In the past decade, whole-genome and whole-exome sequencing studies have identified recurrent genetic alterations with potentially actionable mutation in other genes: *AKAP9, ZEB1* and *ADCK1* (95). Mutations in *NF1, TERT* promoter and *DICER1* have also been found (109).

Genetic alterations suspected to promote angiogenesis have been reported in PCs, suggesting the possibility of using anti-angiogenic drugs to block tumour growth in these patients, based on their PC somatic genetic profile. Variants in the *KDR* gene, encoding the pro-angiogenic VEGF-R2 (vascular endothelial factor receptor 2) were found in 13% of PCs, alongside variants in other genes implicated in angiogenesis regulation such as *EPHB4*, *PTPRB* and *VCAN* (92). The identification of an activating somatic variant in the *KDR* gene justified the employment of VEGFR-targeted drugs cabozantinib and regorafenib (92).

***Other rare somatic mutations*** were reported in PC: *TP53* gene located on chromosome 17p13.1, a tumour suppressor gene that controls and regulates apoptosis (in one of three PC cases) (108). A downregulation of the *TBX1* gene located on chromosome 22q11.21, a candidate gene of DiGeorge syndrome and congenital hypoparathyroidism, has been detected in PC (113).

A recent gene expression study investigating 740 genes implicated in cancer progression related processes was performed in PC from patients with and without metastases. A specific sub-panel of 144 genes that were upregulated (*ANGPTL4, BMP7, CD24, FDFR1, MMP9, SOX2*) and downregulated (*ERBB3, FBP1, RAB25, TBX1*) was identified in metastatic PC. Further studies are needed to better define the possible role of these deregulated genes in the metastatic phenotype of PC (114).

***Mutation signatures*** were investigated searching for DNA repair defects and high tumour mutational burden (TMB). In selected cases with high TMB and APOBEC-associated signatures (62, 92, 115, 116, 127), immune checkpoint blockade (e.g. pembrolizumab) has produced biochemical responses and prolonged disease stabilization (115, 116, 127).

## Epigenetic mechanisms

Epigenetic mechanisms involving modifications in histone methylation and DNA methylation have been reported in PC, revealing up- or downregulation expression of several proteins (117). Promoter of hypermethylation of genes that downregulate cyclin D1 expression and genes associated with silencing of Wnt-pathway have been reported (72, 118, 119).

## MicroRNAs

MicroRNAs (miRNAs) are small (19–25 nucleotides long), non-coding RNAs, that function as negative regulators of gene expression, at the post-transcriptional level, by increasing degradation or decreasing translation of target mRNA. There are both overexpressed miRNAs acting as oncogenes (oncomiR) and silenced, downregulated miRNAs usually acting as tumour suppressors (120, 121). Compared with normal parathyroid tissue, miRNAs behaviour in PCs showed a globally lowered expression. These findings suggest that miRNA expression may contribute to the development of PC (122). These miRNAs not only have intracellular localization but also are present in biological fluids, as circulating miRNAs (c-miRNAs), both as free molecules and within exosomes. A recent study analysed the expression of 754 c-miRNAs in the serum samples from 13 patients with PC and 11 patients with PA, finding 17 c-miRNAs to be significantly downregulated in the PC group, suggesting a potential diagnostic biomarker for differential diagnosis of PC (122).

## Long non-coding RNAs

Long non-coding RNAs (lncRNAs) are non-coding transcripts >200 nucleotides. They function as epigenetic regulators of gene expression, essentially in tissue-specific manner and dysregulated expression of lncRNAs may play a role in cancer biology (123), being responsible for cell viability, invasion and migration (124). Profiling analyses of lncRNA showed down- and upregulation in PC compared to PA and were able to distinguish between the two types of tumours (125). The overexpression of lncRNAs was observed in cases with *CDC73* variants that showed a more aggressive clinical phenotype compared with sporadic PC (126), suggesting a role in PC carcinogenesis and prognosis as an epigenetic modulator and potential biomarker with a potential use in the post-surgery settings for patient follow-up.

## Conclusion

PC continues to be an exceptionally rare malignancy worldwide. Apparent increases in incidence in some regions in the past two decades are primarily due to enhanced detection methods and reporting practices in the healthcare systems. While the absolute number of PC cases has risen, this trend is probably attributable to population growth and survival and improved diagnostic capabilities rather than a significant change in the underlying causation. The increase in incidence of PC has mirrored the increase in incidence of PHPT overall, which was favoured by the introduction of automated chemistry panels for calcium screening in 1974, new IRMA assays for PTH measurement and proactive guidelines for osteoporosis screening and treatment in early 2000. The use of routine neck ultrasonography and the development of ‘minimally invasive’ surgery for parathyroid disease decreased the costs of intervention. Consequently, the referral for parathyroidectomy in asymptomatic disease has increased (13, 26, 27, 28, 29, 30, 31, 32, 33, 34, 35, 36, 37, 38, 39, 40, 41, 42).

Accurate and timely diagnosis of PC is of major importance due to the aggressive behaviour of the tumour and the poor eventual prognosis. Distinction between malignant and benign disease based only on clinical and histological criteria is still extremely challenging. In the past two decades, advances in molecular genetic studies have allowed the identification of specific genetic and epigenetic abnormalities and better characterization of PCs, thereby improving the distinction of PC from benign PA. The genetic aetiology of PC involves many genes, the major driver mutation being germinal and somatic *CDC73* gene variants (63, 64, 65, 66, 67, 68, 69, 70, 71, 72, 73, 74, 75, 76, 77, 78, 81). Other genes, such as *MEN1* (82, 83, 84, 85, 86, 87, 88, 89, 90, 91, 92), *RET* (85, 93, 94) and *PRUNE2* (95, 108); the PI3K/AKT/mTOR pathway (92, 107, 109, 110, 111); and epigenetic mechanisms are involved in the alteration of miRNA expression; however, other as yet unidentified genes and pathways also likely contribute to disease onset and progression (72, 117, 118, 119, 120, 121, 122, 123, 124, 125, 126). Arriving at a diagnosis of PC when definitive evidence of metastasis or local invasion is absent requires a structured approach that combines clinical, hormonal, imaging, pathological and genetic evidence. Such a structured approach may help to avoid pitfalls and decreased overdiagnosis of PC in cases with equivocal supportive evidence.

Inhibitors of PI3K/AKT/mTOR pathway, anti-angiogenic drugs and immune checkpoint blockers have shown promising results in selected PC case reports, in controlling hypercalcaemia and stabilizing or slowing tumour progression. The extreme rarity of PC cases and the large spectrum of genetic and epigenetic alterations identified in affected patients represent the major limitation in the conception of randomized controlled clinical trials to test targeted therapies (92, 108, 109, 112, 115, 116, 127).

## Declaration of interest

The authors declare that there is no conflict of interest that could be perceived as prejudicing the impartiality of the work reported.

## Funding

This work did not receive any specific grant from any funding agency in the public, commercial or not-for-profit sector.
